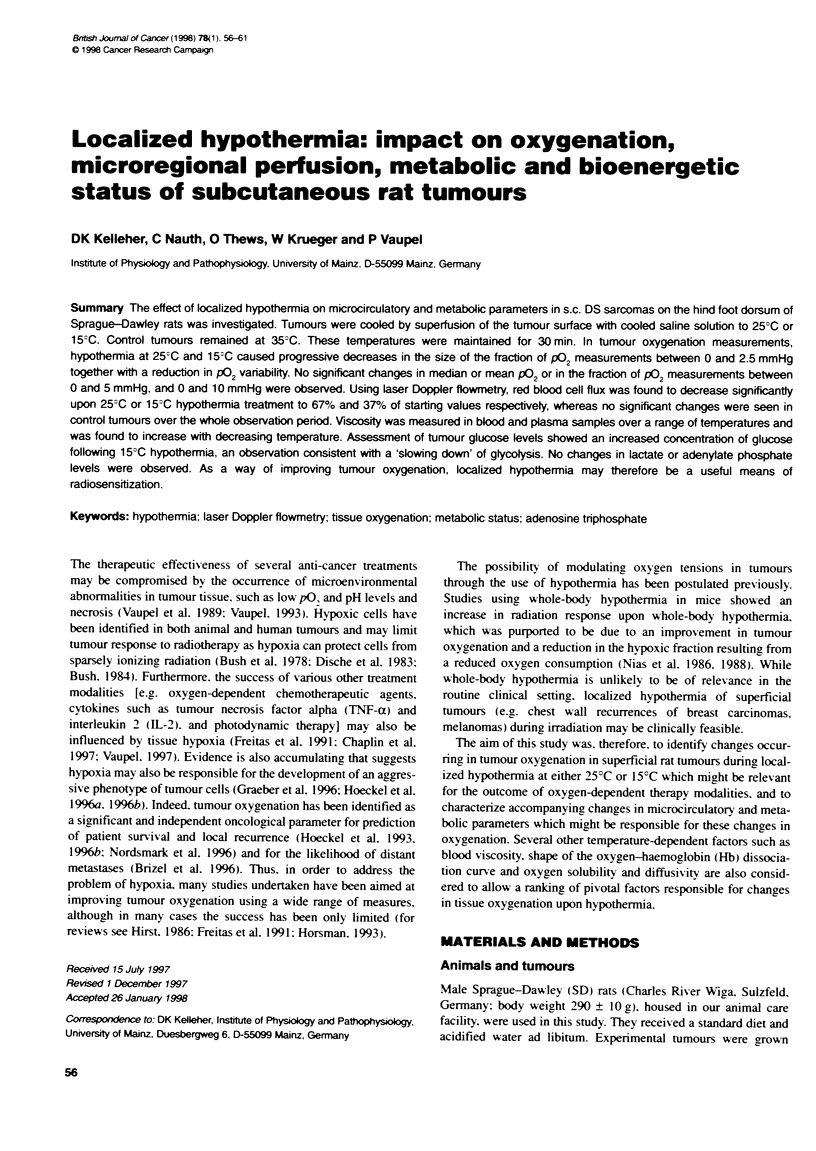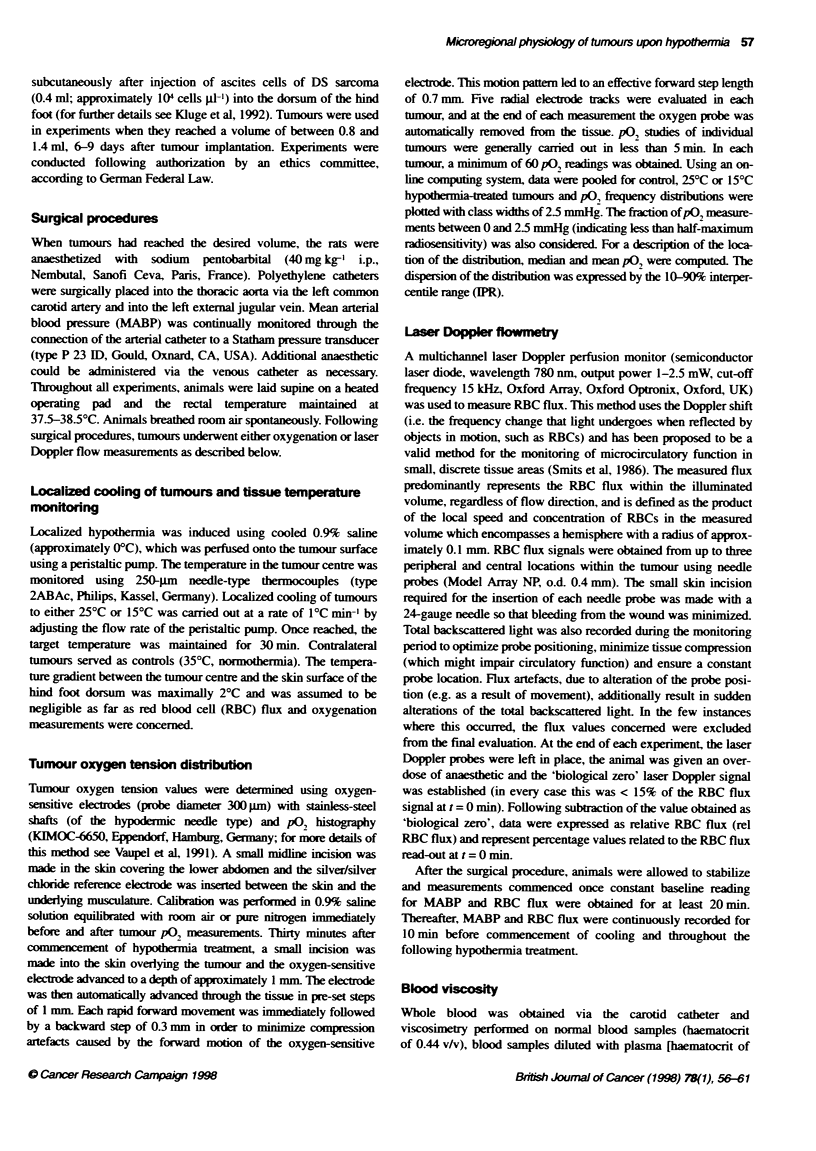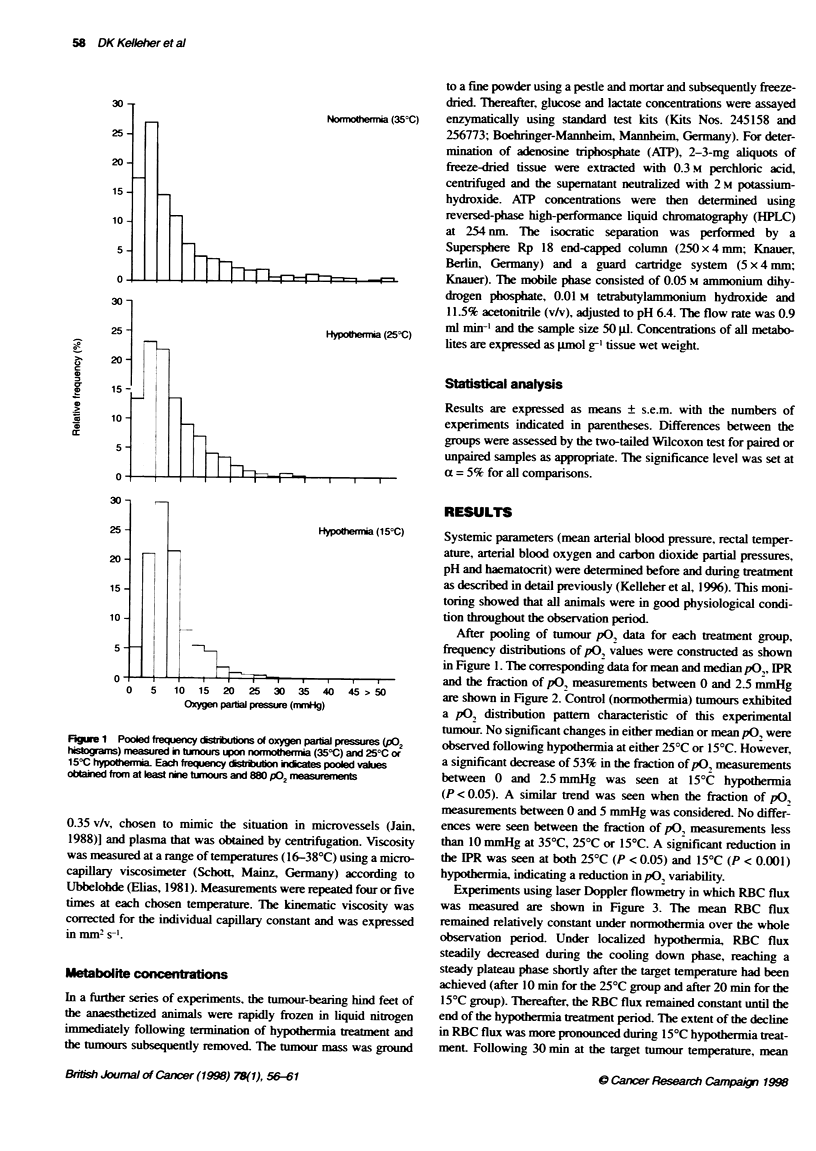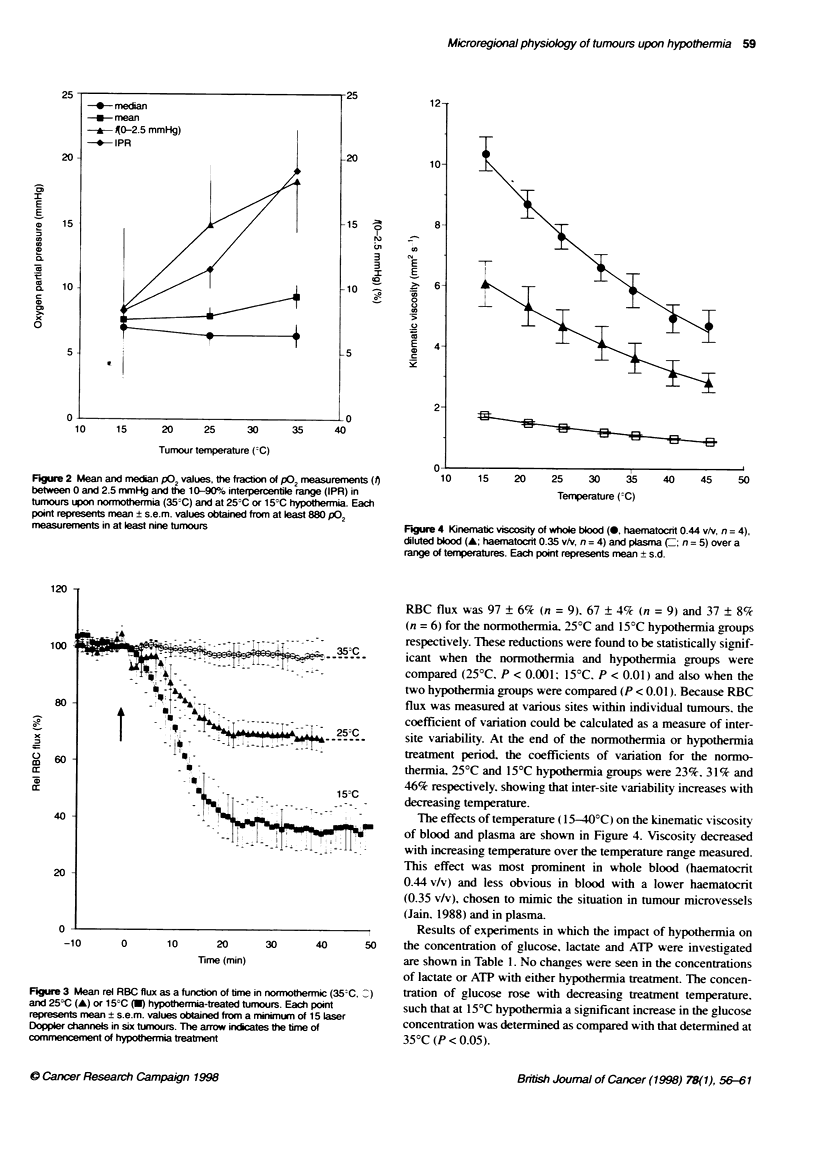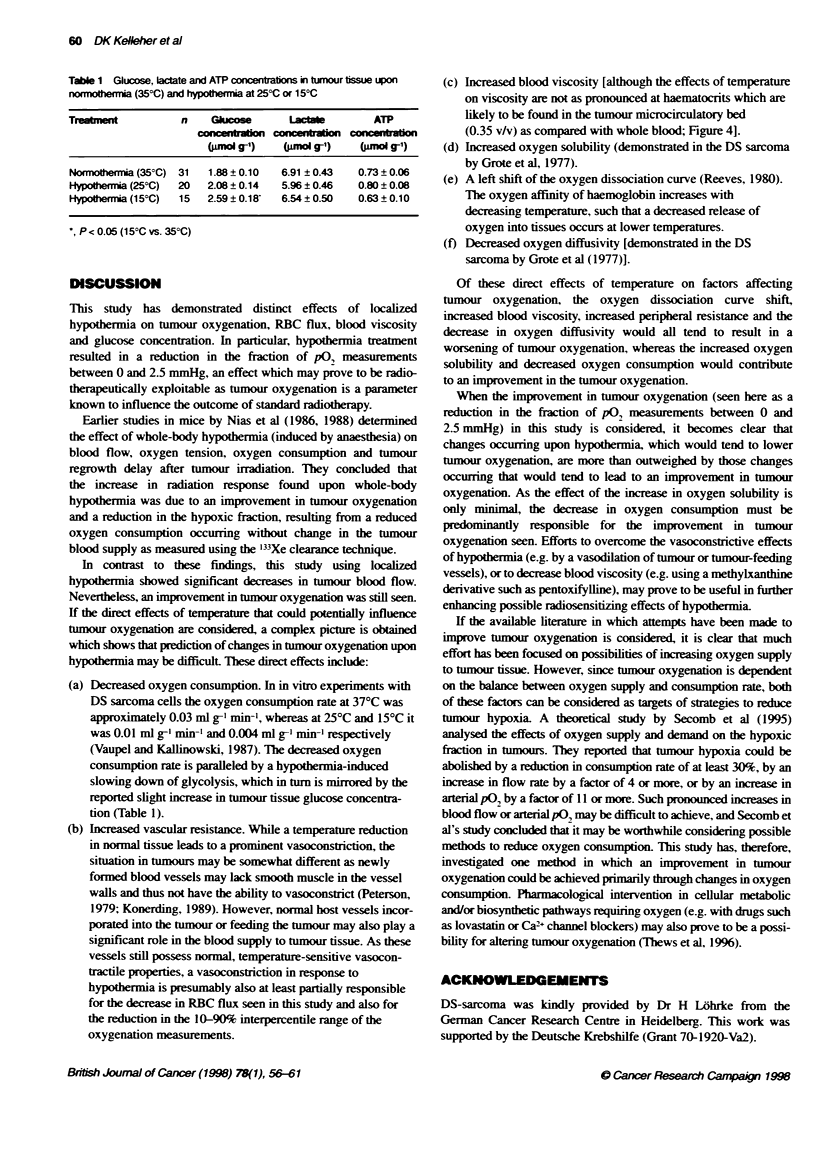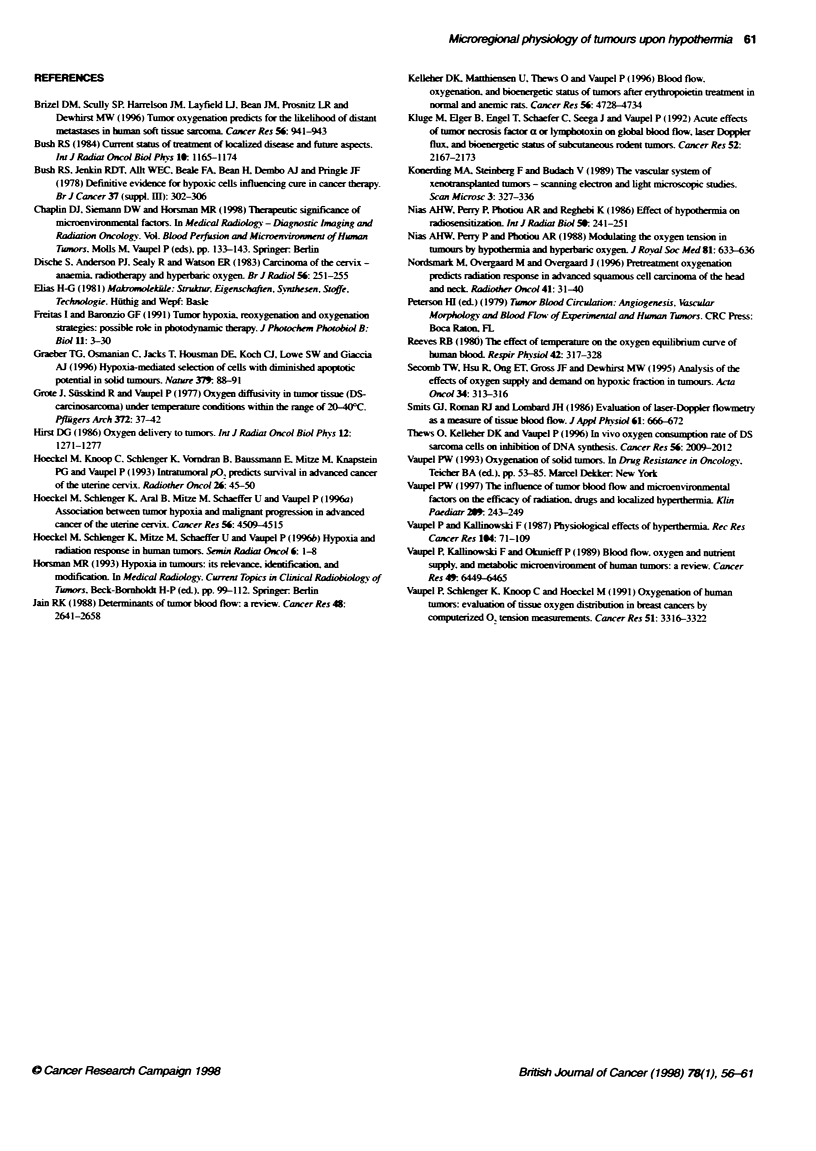# Localized hypothermia: impact on oxygenation, microregional perfusion, metabolic and bioenergetic status of subcutaneous rat tumours.

**DOI:** 10.1038/bjc.1998.442

**Published:** 1998-07

**Authors:** D. K. Kelleher, C. Nauth, O. Thews, W. Krueger, P. Vaupel

**Affiliations:** Institute of Physiology and Pathophysiology, University of Mainz, Germany.

## Abstract

The effect of localized hypothermia on microcirculatory and metabolic parameters in s.c. DS sarcomas on the hind foot dorsum of Sprague-Dawley rats was investigated. Tumours were cooled by superfusion of the tumour surface with cooled saline solution to 25 degrees C or 15 degrees C. Control tumours remained at 35 degrees C. These temperatures were maintained for 30 min. In tumour oxygenation measurements, hypothermia at 25 degrees C and 15 degrees C caused progressive decreases in the size of the fraction of pO2 measurements between 0 and 2.5 mmHg together with a reduction in pO2 variability. No significant changes in median or mean pO2 or in the fraction of pO2 measurements between 0 and 5 mmHg, and 0 and 10 mmHg were observed. Using laser Doppler flowmetry, red blood cell flux was found to decrease significantly upon 25 degrees C or 15 degrees C hypothermia treatment to 67% and 37% of starting values respectively, whereas no significant changes were seen in control tumours over the whole observation period. Viscosity was measured in blood and plasma samples over a range of temperatures and was found to increase with decreasing temperature. Assessment of tumour glucose levels showed an increased concentration of glucose following 15 degrees C hypothermia, an observation consistent with a 'slowing down' of glycolysis. No changes in lactate or adenylate phosphate levels were observed. As a way of improving tumour oxygenation, localized hypothermia may therefore be a useful means of radiosensitization.


British Journal of Cancer (1998) 78(1), 56–61
© 1998 Cancer Research Campaign

### Localized hypothermia: impact on oxygenation, microregional perfusion, metabolic and bioenergetic status of subcutaneous rat tumours

**DK Kelleher, C Nauth, O Thews, W Krueger and P Vaupel**

Institute of Physiology and Pathophysiology, University of Mainz, D-55099 Mainz, Germany

**Summary** The effect of localized hypothermia on microcirculatory and metabolic parameters in s.c. DS sarcomas on the hind foot dorsum of Sprague–Dawley rats was investigated. Tumours were cooled by superfusion of the tumour surface with cooled saline solution to 25°C or 15°C. Control tumours remained at 35°C. These temperatures were maintained for 30 min. In tumour oxygenation measurements, hypothermia at 25°C and 15°C caused progressive decreases in the size of the fraction of $pO_2$ measurements between 0 and 2.5 mmHg together with a reduction in $pO_2$ variability. No significant changes in median or mean $pO_2$ or in the fraction of $pO_2$ measurements between 0 and 5 mmHg, and 0 and 10 mmHg were observed. Using laser Doppler flowmetry, red blood cell flux was found to decrease significantly upon 25°C or 15°C hypothermia treatment to 67% and 37% of starting values respectively, whereas no significant changes were seen in control tumours over the whole observation period. Viscosity was measured in blood and plasma samples over a range of temperatures and was found to increase with decreasing temperature. Assessment of tumour glucose levels showed an increased concentration of glucose following 15°C hypothermia, an observation consistent with a 'slowing down' of glycolysis. No changes in lactate or adenylate phosphate levels were observed. As a way of improving tumour oxygenation, localized hypothermia may therefore be a useful means of radiosensitization.

**Keywords:** hypothermia; laser Doppler flowmetry; tissue oxygenation; metabolic status; adenosine triphosphate

The therapeutic effectiveness of several anti-cancer treatments may be compromised by the occurrence of microenvironmental abnormalities in tumour tissue, such as low $pO_2$ and pH levels and necrosis (Vaupel et al, 1989; Vaupel, 1993). Hypoxic cells have been identified in both animal and human tumours and may limit tumour response to radiotherapy as hypoxia can protect cells from sparsely ionizing radiation (Bush et al, 1978; Dische et al, 1983; Bush, 1984). Furthermore, the success of various other treatment modalities [e.g. oxygen-dependent chemotherapeutic agents, cytokines such as tumour necrosis factor alpha (TNF-α) and interleukin 2 (IL-2), and photodynamic therapy] may also be influenced by tissue hypoxia (Freitas et al, 1991; Chaplin et al, 1997; Vaupel, 1997). Evidence is also accumulating that suggests hypoxia may also be responsible for the development of an aggressive phenotype of tumour cells (Graeber et al, 1996; Hoeckel et al, 1996*a*, 1996*b*). Indeed, tumour oxygenation has been identified as a significant and independent oncological parameter for prediction of patient survival and local recurrence (Hoeckel et al, 1993, 1996*b*; Nordsmark et al, 1996) and for the likelihood of distant metastases (Brizel et al, 1996). Thus, in order to address the problem of hypoxia, many studies undertaken have been aimed at improving tumour oxygenation using a wide range of measures, although in many cases the success has been only limited (for reviews see Hirst, 1986; Freitas et al, 1991; Horsman, 1993).

*Received 15 July 1997*
*Revised 1 December 1997*
*Accepted 26 January 1998*

*Correspondence to:* DK Kelleher, Institute of Physiology and Pathophysiology, University of Mainz, Duesbergweg 6, D-55099 Mainz, Germany

The possibility of modulating oxygen tensions in tumours through the use of hypothermia has been postulated previously. Studies using whole-body hypothermia in mice showed an increase in radiation response upon whole-body hypothermia, which was purported to be due to an improvement in tumour oxygenation and a reduction in the hypoxic fraction resulting from a reduced oxygen consumption (Nias et al, 1986, 1988). While whole-body hypothermia is unlikely to be of relevance in the routine clinical setting, localized hypothermia of superficial tumours (e.g. chest wall recurrences of breast carcinomas, melanomas) during irradiation may be clinically feasible.

The aim of this study was, therefore, to identify changes occurring in tumour oxygenation in superficial rat tumours during localized hypothermia at either 25°C or 15°C which might be relevant for the outcome of oxygen-dependent therapy modalities, and to characterize accompanying changes in microcirculatory and metabolic parameters which might be responsible for these changes in oxygenation. Several other temperature-dependent factors such as blood viscosity, shape of the oxygen–haemoglobin (Hb) dissociation curve and oxygen solubility and diffusivity are also considered to allow a ranking of pivotal factors responsible for changes in tissue oxygenation upon hypothermia.

### MATERIALS AND METHODS

### Animals and tumours

Male Sprague–Dawley (SD) rats (Charles River Wiga, Sulzfeld, Germany; body weight 290 ± 10 g), housed in our animal care facility, were used in this study. They received a standard diet and acidified water ad libitum. Experimental tumours were grown subcutaneously after injection of ascites cells of DS sarcoma (0.4 ml; approximately $10^4$ cells $\mu l^{-1}$) into the dorsum of the hind foot (for further details see Kluge et al, 1992). Tumours were used in experiments when they reached a volume of between 0.8 and 1.4 ml, 6–9 days after tumour implantation. Experiments were conducted following authorization by an ethics committee, according to German Federal Law.

### Surgical procedures

When tumours had reached the desired volume, the rats were anaesthetized with sodium pentobarbital (40 mg $kg^{-1}$ i.p., Nembutal, Sanofi Ceva, Paris, France). Polyethylene catheters were surgically placed into the thoracic aorta via the left common carotid artery and into the left external jugular vein. Mean arterial blood pressure (MABP) was continually monitored through the connection of the arterial catheter to a Statham pressure transducer (type P 23 ID, Gould, Oxnard, CA, USA). Additional anaesthetic could be administered via the venous catheter as necessary. Throughout all experiments, animals were laid supine on a heated operating pad and the rectal temperature maintained at 37.5–38.5°C. Animals breathed room air spontaneously. Following surgical procedures, tumours underwent either oxygenation or laser Doppler flow measurements as described below.

### Localized cooling of tumours and tissue temperature monitoring

Localized hypothermia was induced using cooled 0.9% saline (approximately 0°C), which was perfused onto the tumour surface using a peristaltic pump. The temperature in the tumour centre was monitored using 250-µm needle-type thermocouples (type 2ABAc, Philips, Kassel, Germany). Localized cooling of tumours to either 25°C or 15°C was carried out at a rate of 1°C $min^{-1}$ by adjusting the flow rate of the peristaltic pump. Once reached, the target temperature was maintained for 30 min. Contralateral tumours served as controls (35°C, normothermia). The temperature gradient between the tumour centre and the skin surface of the hind foot dorsum was maximally 2°C and was assumed to be negligible as far as red blood cell (RBC) flux and oxygenation measurements were concerned.

### Tumour oxygen tension distribution

Tumour oxygen tension values were determined using oxygen-sensitive electrodes (probe diameter 300 µm) with stainless-steel shafts (of the hypodermic needle type) and $pO_2$ histography (KIMOC-6650, Eppendorf, Hamburg, Germany; for more details of this method see Vaupel et al, 1991). A small midline incision was made in the skin covering the lower abdomen and the silver/silver chloride reference electrode was inserted between the skin and the underlying musculature. Calibration was performed in 0.9% saline solution equilibrated with room air or pure nitrogen immediately before and after tumour $pO_2$ measurements. Thirty minutes after commencement of hypothermia treatment, a small incision was made into the skin overlying the tumour and the oxygen-sensitive electrode advanced to a depth of approximately 1 mm. The electrode was then automatically advanced through the tissue in pre-set steps of 1 mm. Each rapid forward movement was immediately followed by a backward step of 0.3 mm in order to minimize compression artefacts caused by the forward motion of the oxygen-sensitive electrode. This motion pattern led to an effective forward step length of 0.7 mm. Five radial electrode tracks were evaluated in each tumour, and at the end of each measurement the oxygen probe was automatically removed from the tissue. $pO_2$ studies of individual tumours were generally carried out in less than 5 min. In each tumour, a minimum of 60 $pO_2$ readings was obtained. Using an online computing system, data were pooled for control, 25°C or 15°C hypothermia-treated tumours and $pO_2$ frequency distributions were plotted with class widths of 2.5 mmHg. The fraction of $pO_2$ measurements between 0 and 2.5 mmHg (indicating less than half-maximum radiosensitivity) was also considered. For a description of the location of the distribution, median and mean $pO_2$ were computed. The dispersion of the distribution was expressed by the 10–90% interpercentile range (IPR).

### Laser Doppler flowmetry

A multichannel laser Doppler perfusion monitor (semiconductor laser diode, wavelength 780 nm, output power 1–2.5 mW, cut-off frequency 15 kHz, Oxford Array, Oxford Optronix, Oxford, UK) was used to measure RBC flux. This method uses the Doppler shift (i.e. the frequency change that light undergoes when reflected by objects in motion, such as RBCs) and has been proposed to be a valid method for the monitoring of microcirculatory function in small, discrete tissue areas (Smits et al, 1986). The measured flux predominantly represents the RBC flux within the illuminated volume, regardless of flow direction, and is defined as the product of the local speed and concentration of RBCs in the measured volume which encompasses a hemisphere with a radius of approximately 0.1 mm. RBC flux signals were obtained from up to three peripheral and central locations within the tumour using needle probes (Model Array NP, o.d. 0.4 mm). The small skin incision required for the insertion of each needle probe was made with a 24-gauge needle so that bleeding from the wound was minimized. Total backscattered light was also recorded during the monitoring period to optimize probe positioning, minimize tissue compression (which might impair circulatory function) and ensure a constant probe location. Flux artefacts, due to alteration of the probe position (e.g. as a result of movement), additionally result in sudden alterations of the total backscattered light. In the few instances where this occurred, the flux values concerned were excluded from the final evaluation. At the end of each experiment, the laser Doppler probes were left in place, the animal was given an overdose of anaesthetic and the 'biological zero' laser Doppler signal was established (in every case this was < 15% of the RBC flux signal at $t = 0$ min). Following subtraction of the value obtained as 'biological zero', data were expressed as relative RBC flux (rel RBC flux) and represent percentage values related to the RBC flux read-out at $t = 0$ min.

After the surgical procedure, animals were allowed to stabilize and measurements commenced once constant baseline reading for MABP and RBC flux were obtained for at least 20 min. Thereafter, MABP and RBC flux were continuously recorded for 10 min before commencement of cooling and throughout the following hypothermia treatment.

### Blood viscosity

Whole blood was obtained via the carotid catheter and viscosimetry performed on normal blood samples (haematocrit of 0.44 v/v), blood samples diluted with plasma [haematocrit of

© Cancer Research Campaign 1998 

**Figure 1** Pooled frequency distributions of oxygen partial pressures ($pO_2$ histograms) measured in tumours upon normothermia (35°C) and 25°C or 15°C hypothermia. Each frequency distribution indicates pooled values obtained from at least nine tumours and 880 $pO_2$ measurements

0.35 v/v, chosen to mimic the situation in microvessels (Jain, 1988)] and plasma that was obtained by centrifugation. Viscosity was measured at a range of temperatures (16–38°C) using a micro-capillary viscosimeter (Schott, Mainz, Germany) according to Ubbelohde (Elias, 1981). Measurements were repeated four or five times at each chosen temperature. The kinematic viscosity was corrected for the individual capillary constant and was expressed in $mm^2 s^{-1}$.

### Metabolite concentrations

In a further series of experiments, the tumour-bearing hind feet of the anaesthetized animals were rapidly frozen in liquid nitrogen immediately following termination of hypothermia treatment and the tumours subsequently removed. The tumour mass was ground to a fine powder using a pestle and mortar and subsequently freeze-dried. Thereafter, glucose and lactate concentrations were assayed enzymatically using standard test kits (Kits Nos. 245158 and 256773; Boehringer-Mannheim, Mannheim, Germany). For determination of adenosine triphosphate (ATP), 2–3-mg aliquots of freeze-dried tissue were extracted with 0.3 M perchloric acid, centrifuged and the supernatant neutralized with 2 M potassium-hydroxide. ATP concentrations were then determined using reversed-phase high-performance liquid chromatography (HPLC) at 254 nm. The isocratic separation was performed by a Supersphere Rp 18 end-capped column (250 × 4 mm; Knauer, Berlin, Germany) and a guard cartridge system (5 × 4 mm; Knauer). The mobile phase consisted of 0.05 M ammonium dihydrogen phosphate, 0.01 M tetrabutylammonium hydroxide and 11.5% acetonitrile (v/v), adjusted to pH 6.4. The flow rate was 0.9 ml $min^{-1}$ and the sample size 50 μl. Concentrations of all metabolites are expressed as μmol $g^{-1}$ tissue wet weight.

### Statistical analysis

Results are expressed as means ± s.e.m. with the numbers of experiments indicated in parentheses. Differences between the groups were assessed by the two-tailed Wilcoxon test for paired or unpaired samples as appropriate. The significance level was set at $\alpha = 5\%$ for all comparisons.

### RESULTS

Systemic parameters (mean arterial blood pressure, rectal temperature, arterial blood oxygen and carbon dioxide partial pressures, pH and haematocrit) were determined before and during treatment as described in detail previously (Kelleher et al, 1996). This monitoring showed that all animals were in good physiological condition throughout the observation period.

After pooling of tumour $pO_2$ data for each treatment group, frequency distributions of $pO_2$ values were constructed as shown in Figure 1. The corresponding data for mean and median $pO_2$, IPR and the fraction of $pO_2$ measurements between 0 and 2.5 mmHg are shown in Figure 2. Control (normothermia) tumours exhibited a $pO_2$ distribution pattern characteristic of this experimental tumour. No significant changes in either median or mean $pO_2$ were observed following hypothermia at either 25°C or 15°C. However, a significant decrease of 53% in the fraction of $pO_2$ measurements between 0 and 2.5 mmHg was seen at 15°C hypothermia ($P < 0.05$). A similar trend was seen when the fraction of $pO_2$ measurements between 0 and 5 mmHg was considered. No differences were seen between the fraction of $pO_2$ measurements less than 10 mmHg at 35°C, 25°C or 15°C. A significant reduction in the IPR was seen at both 25°C ($P < 0.05$) and 15°C ($P < 0.001$) hypothermia, indicating a reduction in $pO_2$ variability.

Experiments using laser Doppler flowmetry in which RBC flux was measured are shown in Figure 3. The mean RBC flux remained relatively constant under normothermia over the whole observation period. Under localized hypothermia, RBC flux steadily decreased during the cooling down phase, reaching a steady plateau phase shortly after the target temperature had been achieved (after 10 min for the 25°C group and after 20 min for the 15°C group). Thereafter, the RBC flux remained constant until the end of the hypothermia treatment period. The extent of the decline in RBC flux was more pronounced during 15°C hypothermia treatment. Following 30 min at the target tumour temperature, mean

© Cancer Research Campaign 1998

**Figure 2** Mean and median $pO_2$ values, the fraction of $pO_2$ measurements ($f$) between 0 and 2.5 mmHg and the 10–90% interpercentile range (IPR) in tumours upon normothermia (35°C) and at 25°C or 15°C hypothermia. Each point represents mean ± s.e.m. values obtained from at least 880 $pO_2$ measurements in at least nine tumours

**Figure 4** Kinematic viscosity of whole blood (●, haematocrit 0.44 v/v, $n$ = 4), diluted blood (▲; haematocrit 0.35 v/v, $n$ = 4) and plasma (□; $n$ = 5) over a range of temperatures. Each point represents mean ± s.d.

**Figure 3** Mean rel RBC flux as a function of time in normothermic (35°C, ○) and 25°C (▲) or 15°C (■) hypothermia-treated tumours. Each point represents mean ± s.e.m. values obtained from a minimum of 15 laser Doppler channels in six tumours. The arrow indicates the time of commencement of hypothermia treatment

RBC flux was 97 ± 6% ($n$ = 9), 67 ± 4% ($n$ = 9) and 37 ± 8% ($n$ = 6) for the normothermia, 25°C and 15°C hypothermia groups respectively. These reductions were found to be statistically significant when the normothermia and hypothermia groups were compared (25°C, $P < 0.001$; 15°C, $P < 0.01$) and also when the two hypothermia groups were compared ($P < 0.01$). Because RBC flux was measured at various sites within individual tumours, the coefficient of variation could be calculated as a measure of inter-site variability. At the end of the normothermia or hypothermia treatment period, the coefficients of variation for the normothermia, 25°C and 15°C hypothermia groups were 23%, 31% and 46% respectively, showing that inter-site variability increases with decreasing temperature.

The effects of temperature (15–40°C) on the kinematic viscosity of blood and plasma are shown in Figure 4. Viscosity decreased with increasing temperature over the temperature range measured. This effect was most prominent in whole blood (haematocrit 0.44 v/v) and less obvious in blood with a lower haematocrit (0.35 v/v), chosen to mimic the situation in tumour microvessels (Jain, 1988) and in plasma.

Results of experiments in which the impact of hypothermia on the concentration of glucose, lactate and ATP were investigated are shown in Table 1. No changes were seen in the concentrations of lactate or ATP with either hypothermia treatment. The concentration of glucose rose with decreasing treatment temperature, such that at 15°C hypothermia a significant increase in the glucose concentration was determined as compared with that determined at 35°C ($P < 0.05$).

© Cancer Research Campaign 1998

Table 1 Glucose, lactate and ATP concentrations in tumour tissue upon normothermia (35°C) and hypothermia at 25°C or 15°C

| Treatment | n | Glucose concentration (μmol g⁻¹) | Lactate concentration (μmol g⁻¹) | ATP concentration (μmol g⁻¹) |
|---|---|---|---|---|
| Normothermia (35°C) | 31 | 1.88 ± 0.10 | 6.91 ± 0.43 | 0.73 ± 0.06 |
| Hypothermia (25°C) | 20 | 2.08 ± 0.14 | 5.96 ± 0.46 | 0.80 ± 0.08 |
| Hypothermia (15°C) | 15 | 2.59 ± 0.18* | 6.54 ± 0.50 | 0.63 ± 0.10 |

*, $P < 0.05$ (15°C vs. 35°C)

### DISCUSSION

This study has demonstrated distinct effects of localized hypothermia on tumour oxygenation, RBC flux, blood viscosity and glucose concentration. In particular, hypothermia treatment resulted in a reduction in the fraction of $pO_2$ measurements between 0 and 2.5 mmHg, an effect which may prove to be radiotherapeutically exploitable as tumour oxygenation is a parameter known to influence the outcome of standard radiotherapy.

Earlier studies in mice by Nias et al (1986, 1988) determined the effect of whole-body hypothermia (induced by anaesthesia) on blood flow, oxygen tension, oxygen consumption and tumour regrowth delay after tumour irradiation. They concluded that the increase in radiation response found upon whole-body hypothermia was due to an improvement in tumour oxygenation and a reduction in the hypoxic fraction, resulting from a reduced oxygen consumption occurring without change in the tumour blood supply as measured using the $^{133}Xe$ clearance technique.

In contrast to these findings, this study using localized hypothermia showed significant decreases in tumour blood flow. Nevertheless, an improvement in tumour oxygenation was still seen. If the direct effects of temperature that could potentially influence tumour oxygenation are considered, a complex picture is obtained which shows that prediction of changes in tumour oxygenation upon hypothermia may be difficult. These direct effects include:

(a) Decreased oxygen consumption. In in vitro experiments with DS sarcoma cells the oxygen consumption rate at 37°C was approximately 0.03 ml $g^{-1}$ $min^{-1}$, whereas at 25°C and 15°C it was 0.01 ml $g^{-1}$ $min^{-1}$ and 0.004 ml $g^{-1}$ $min^{-1}$ respectively (Vaupel and Kallinowski, 1987). The decreased oxygen consumption rate is paralleled by a hypothermia-induced slowing down of glycolysis, which in turn is mirrored by the reported slight increase in tumour tissue glucose concentration (Table 1).

(b) Increased vascular resistance. While a temperature reduction in normal tissue leads to a prominent vasoconstriction, the situation in tumours may be somewhat different as newly formed blood vessels may lack smooth muscle in the vessel walls and thus not have the ability to vasoconstrict (Peterson, 1979; Konerding, 1989). However, normal host vessels incorporated into the tumour or feeding the tumour may also play a significant role in the blood supply to tumour tissue. As these vessels still possess normal, temperature-sensitive vasocontractile properties, a vasoconstriction in response to hypothermia is presumably also at least partially responsible for the decrease in RBC flux seen in this study and also for the reduction in the 10–90% interpercentile range of the oxygenation measurements.

(c) Increased blood viscosity [although the effects of temperature on viscosity are not as pronounced at haematocrits which are likely to be found in the tumour microcirculatory bed (0.35 v/v) as compared with whole blood; Figure 4].

(d) Increased oxygen solubility (demonstrated in the DS sarcoma by Grote et al, 1977).

(e) A left shift of the oxygen dissociation curve (Reeves, 1980). The oxygen affinity of haemoglobin increases with decreasing temperature, such that a decreased release of oxygen into tissues occurs at lower temperatures.

(f) Decreased oxygen diffusivity [demonstrated in the DS sarcoma by Grote et al (1977)].

Of these direct effects of temperature on factors affecting tumour oxygenation, the oxygen dissociation curve shift, increased blood viscosity, increased peripheral resistance and the decrease in oxygen diffusivity would all tend to result in a worsening of tumour oxygenation, whereas the increased oxygen solubility and decreased oxygen consumption would contribute to an improvement in the tumour oxygenation.

When the improvement in tumour oxygenation (seen here as a reduction in the fraction of $pO_2$ measurements between 0 and 2.5 mmHg) in this study is considered, it becomes clear that changes occurring upon hypothermia, which would tend to lower tumour oxygenation, are more than outweighed by those changes occurring that would tend to lead to an improvement in tumour oxygenation. As the effect of the increase in oxygen solubility is only minimal, the decrease in oxygen consumption must be predominantly responsible for the improvement in tumour oxygenation seen. Efforts to overcome the vasoconstrictive effects of hypothermia (e.g. by a vasodilation of tumour or tumour-feeding vessels), or to decrease blood viscosity (e.g. using a methylxanthine derivative such as pentoxifylline), may prove to be useful in further enhancing possible radiosensitizing effects of hypothermia.

If the available literature in which attempts have been made to improve tumour oxygenation is considered, it is clear that much effort has been focused on possibilities of increasing oxygen supply to tumour tissue. However, since tumour oxygenation is dependent on the balance between oxygen supply and consumption rate, both of these factors can be considered as targets of strategies to reduce tumour hypoxia. A theoretical study by Secomb et al (1995) analysed the effects of oxygen supply and demand on the hypoxic fraction in tumours. They reported that tumour hypoxia could be abolished by a reduction in consumption rate of at least 30%, by an increase in flow rate by a factor of 4 or more, or by an increase in arterial $pO_2$ by a factor of 11 or more. Such pronounced increases in blood flow or arterial $pO_2$ may be difficult to achieve, and Secomb et al's study concluded that it may be worthwhile considering possible methods to reduce oxygen consumption. This study has, therefore, investigated one method in which an improvement in tumour oxygenation could be achieved primarily through changes in oxygen consumption. Pharmacological intervention in cellular metabolic and/or biosynthetic pathways requiring oxygen (e.g. with drugs such as lovastatin or $Ca^{2+}$ channel blockers) may also prove to be a possibility for altering tumour oxygenation (Thews et al, 1996).

### ACKNOWLEDGEMENTS

DS-sarcoma was kindly provided by Dr H Löhrke from the German Cancer Research Centre in Heidelberg. This work was supported by the Deutsche Krebshilfe (Grant 70-1920-Va2).

© Cancer Research Campaign 1998

### REFERENCES

Brizel DM, Scully SP, Harrelson JM, Layfield LJ, Bean JM, Prosnitz LR and Dewhirst MW (1996) Tumor oxygenation predicts for the likelihood of distant metastases in human soft tissue sarcoma. *Cancer Res* **56**: 941–943

Bush RS (1984) Current status of treatment of localized disease and future aspects. *Int J Radiat Oncol Biol Phys* **10**: 1165–1174

Bush RS, Jenkin RDT, Allt WEC, Beale FA, Bean H, Dembo AJ and Pringle JF (1978) Definitive evidence for hypoxic cells influencing cure in cancer therapy. *Br J Cancer* **37** (suppl. III): 302–306

Chaplin DJ, Siemann DW and Horsman MR (1998) Therapeutic significance of microenvironmental factors. In *Medical Radiology – Diagnostic Imaging and Radiation Oncology*. Vol. *Blood Perfusion and Microenvironment of Human Tumors*, Molls M, Vaupel P (eds), pp. 133–143. Springer: Berlin

Dische S, Anderson PJ, Sealy R and Watson ER (1983) Carcinoma of the cervix – anaemia, radiotherapy and hyperbaric oxygen. *Br J Radiol* **56**: 251–255

Elias H-G (1981) *Makromoleküle: Struktur, Eigenschaften, Synthesen, Stoffe, Technologie*. Hüthig and Wepf: Basle

Freitas I and Baronzio GF (1991) Tumor hypoxia, reoxygenation and oxygenation strategies: possible role in photodynamic therapy. *J Photochem Photobiol B: Biol* **11**: 3–30

Graeber TG, Osmanian C, Jacks T, Housman DE, Koch CJ, Lowe SW and Giaccia AJ (1996) Hypoxia-mediated selection of cells with diminished apoptotic potential in solid tumours. *Nature* **379**: 88–91

Grote J, Süsskind R and Vaupel P (1977) Oxygen diffusivity in tumor tissue (DS-carcinosarcoma) under temperature conditions within the range of 20–40°C. *Pflügers Arch* **372**: 37–42

Hirst DG (1986) Oxygen delivery to tumors. *Int J Radiat Oncol Biol Phys* **12**: 1271–1277

Hoeckel M, Knoop C, Schlenger K, Vorndran B, Baussmann E, Mitze M, Knapstein PG and Vaupel P (1993) Intratumoral $pO_2$ predicts survival in advanced cancer of the uterine cervix. *Radiother Oncol* **26**: 45–50

Hoeckel M, Schlenger K, Aral B, Mitze M, Schaeffer U and Vaupel P (1996*a*) Association between tumor hypoxia and malignant progression in advanced cancer of the uterine cervix. *Cancer Res* **56**: 4509–4515

Hoeckel M, Schlenger K, Mitze M, Schaeffer U and Vaupel P (1996*b*) Hypoxia and radiation response in human tumors. *Semin Radiat Oncol* **6**: 1–8

Horsman MR (1993) Hypoxia in tumours: its relevance, identification, and modification. In *Medical Radiology. Current Topics in Clinical Radiobiology of Tumors*, Beck-Bornholdt H-P (ed.), pp. 99–112. Springer: Berlin

Jain RK (1988) Determinants of tumor blood flow: a review. *Cancer Res* **48**: 2641–2658

Kelleher DK, Matthiensen U, Thews O and Vaupel P (1996) Blood flow, oxygenation, and bioenergetic status of tumors after erythropoietin treatment in normal and anemic rats. *Cancer Res* **56**: 4728–4734

Kluge M, Elger B, Engel T, Schaefer C, Seega J and Vaupel P (1992) Acute effects of tumor necrosis factor α or lymphotoxin on global blood flow, laser Doppler flux, and bioenergetic status of subcutaneous rodent tumors. *Cancer Res* **52**: 2167–2173

Konerding MA, Steinberg F and Budach V (1989) The vascular system of xenotransplanted tumors – scanning electron and light microscopic studies. *Scan Microsc* **3**: 327–336

Nias AHW, Perry P, Photiou AR and Reghebi K (1986) Effect of hypothermia on radiosensitization. *Int J Radiat Biol* **50**: 241–251

Nias AHW, Perry P and Photiou AR (1988) Modulating the oxygen tension in tumours by hypothermia and hyperbaric oxygen. *J Royal Soc Med* **81**: 633–636

Nordsmark M, Overgaard M and Overgaard J (1996) Pretreatment oxygenation predicts radiation response in advanced squamous cell carcinoma of the head and neck. *Radiother Oncol* **41**: 31–40

Peterson HI (ed.) (1979) *Tumor Blood Circulation: Angiogenesis, Vascular Morphology and Blood Flow of Experimental and Human Tumors*. CRC Press: Boca Raton, FL

Reeves RB (1980) The effect of temperature on the oxygen equilibrium curve of human blood. *Respir Physiol* **42**: 317–328

Secomb TW, Hsu R, Ong ET, Gross JF and Dewhirst MW (1995) Analysis of the effects of oxygen supply and demand on hypoxic fraction in tumours. *Acta Oncol* **34**: 313–316

Smits GJ, Roman RJ and Lombard JH (1986) Evaluation of laser-Doppler flowmetry as a measure of tissue blood flow. *J Appl Physiol* **61**: 666–672

Thews O, Kelleher DK and Vaupel P (1996) In vivo oxygen consumption rate of DS sarcoma cells on inhibition of DNA synthesis. *Cancer Res* **56**: 2009–2012

Vaupel PW (1993) Oxygenation of solid tumors. In *Drug Resistance in Oncology*, Teicher BA (ed.), pp. 53–85. Marcel Dekker: New York

Vaupel PW (1997) The influence of tumor blood flow and microenvironmental factors on the efficacy of radiation, drugs and localized hyperthermia. *Klin Paediatr* **209**: 243–249

Vaupel P and Kallinowski F (1987) Physiological effects of hyperthermia. *Rec Res Cancer Res* **104**: 71–109

Vaupel P, Kallinowski F and Okunieff P (1989) Blood flow, oxygen and nutrient supply, and metabolic microenvironment of human tumors: a review. *Cancer Res* **49**: 6449–6465

Vaupel P, Schlenger K, Knoop C and Hoeckel M (1991) Oxygenation of human tumors: evaluation of tissue oxygen distribution in breast cancers by computerized $O_2$ tension measurements. *Cancer Res* **51**: 3316–3322

© Cancer Research Campaign 1998